# Optimizing wild bird fecal surveillance sites for HPAI through risk estimation using machine learning algorithms

**DOI:** 10.1016/j.onehlt.2026.101457

**Published:** 2026-05-27

**Authors:** SunMin Kim, Hyunjun Cho, Hyesung Jeong, Dae Sung Yoo, Kyuyoung Lee, Hu Suk Lee, Kyung-Duk Min

**Affiliations:** a College of Veterinary Medicine, Chungbuk National University, Cheongju-si, Chungcheongbuk-do, Republic of Korea; b Wildlife Disease Research Team, National Institute of Wildlife Disease Control and Prevention, Gwangju, Republic of Korea; c Department of Veterinary Epidemiology, College of Veterinary Medicine, Chonnam National University, Gwangju, Republic of Korea; d AI-Bio Convergence Research Institute, Soongsil University, Seoul, Republic of Korea; e College of Veterinary Medicine, Chungnam National University, Daejeon, Republic of Korea

**Keywords:** Avian influenza, Wild bird, Fecal surveillance, Risk map, Republic of Korea

## Abstract

**Background:**

Wild birds are major reservoirs contributing to the spread of highly pathogenic avian influenza (HPAI). Accordingly, fecal-based surveillance of wild birds has been implemented as an early warning system for poultry in South Korea. However, the efficiency of the current system remains limited due to the absence of a quantitative basis. This study aimed to develop a risk-based strategy for optimizing fecal sampling sites, thereby strengthening early warning capacity and preventing the introduction of HPAI into poultry farms.

**Methods:**

The study area comprised 110 designated surveillance areas divided into 1-km edge hexagonal grids. The study period covered October to February from 2021 to 2024. For each grid, we integrated wild bird fecal sampling data with various categories of factors. The dependent variable was the detection of avian influenza virus (AIV) in wild bird feces, while all other variables served as predictors. The risk of AIV detection was estimated using machine learning algorithms, including logistic regression, gradient boosting machine, extreme gradient boosting, and an ensemble model. Model performance was evaluated using the area under the curve (AUC), sensitivity, specificity, and accuracy.

**Results:**

The ensemble model achieved the best predictive performance. Climatic factors, sample size, population, and elevation were identified as the most influential predictors. Spatial inconsistency was observed between the current sampling sites and the proposed risk-based sampling sites.

**Conclusions:**

The proposed risk-based framework enables prioritization of sampling sites, thereby optimizing fecal-based surveillance for HPAI. This approach is expected to enhance the efficiency of EWS and contribute to the prevention of HPAI spread to poultry.

## 1 Introduction

Migratory birds play a critical role as natural reservoirs in the transboundary and international transmission of highly pathogenic avian influenza (HPAI). Although South Korea was designated as an HPAI-free country by the World Organization for Animal Health in 2004, outbreaks subsequently re-emerged [1]. The H5N1 virus recently detected in South Korea may have resulted from reassortment between HPAI viruses that circulated in Europe and North America in 2022 and low-pathogenic avian influenza (LPAI) viruses found in wild birds [2], [3], [4]. Migratory birds infected with these reassorted viruses at Siberian breeding sites may have introduced the virus into South Korea during their overwintering migration [2], [3], [4]. These birds could then have acted as a source of infection for domestic poultry through indirect transmission pathways involving contact with humans, vehicles, or other wildlife [5].

Monitoring HPAI prevalence among wild birds using fecal samples is a widely adopted surveillance strategy, providing early warning for timely interventions to prevent the introduction of HPAI into domestic poultry populations. Fecal surveillance offers several practical advantages. First, samples can be collected non-invasively from the environment, thereby avoiding the animal ethics issues associated with the direct capture of wild birds [6], [7], [8]. Second, fecal surveillance allows for intuitive geographic risk assessment because sampling sites are explicitly recorded. Third, DNA extracted from feces enables the identification of bird species, providing valuable insights into potential virus spread patterns [9]. Owing to these advantages, several countries, including the United States and Germany, have implemented fecal-based monitoring programs [6], [10], [11], [12].

Despite its benefits, the effectiveness of fecal surveillance as an early warning system (EWS) depends on spatial prioritization of sampling sites. Simple random selection without spatial prioritization risks omitting areas with a high risk of HPAI virus detection, which are critical for EWS performance [13]. In contrast, a risk-based strategy that prioritizes high-risk sites provides a rational basis for determining the sequence of sampling [14]. Risk-based approaches have been successfully applied in EWS for other infectious diseases, including Rift Valley fever, West Nile virus, and Zika virus [14]. Furthermore, Cuba has implemented a risk-based surveillance and control system for avian influenza in high-risk poultry populations [15].

Few studies, however, have focused on enhancing wild bird fecal surveillance to support the prevention of HPAI outbreaks in poultry farms. In this context, our study proposes a strategy to optimize fecal sampling sites by incorporating predicted risk as a component of an improved EWS for HPAI in poultry farms in South Korea. To achieve this, we leverage a high-quality national database generated through South Korea's longstanding wild bird fecal surveillance program, which provides detailed information on sample size, virus detection results, and sampling sites. We anticipate that this quantitative framework will improve both the sensitivity and timeliness of EWS and contribute to evidence-based decision-making for preparedness against potential HPAI outbreaks in poultry [16].

## 2 Methods

### 2.1 Study design

**Fig. 1:**
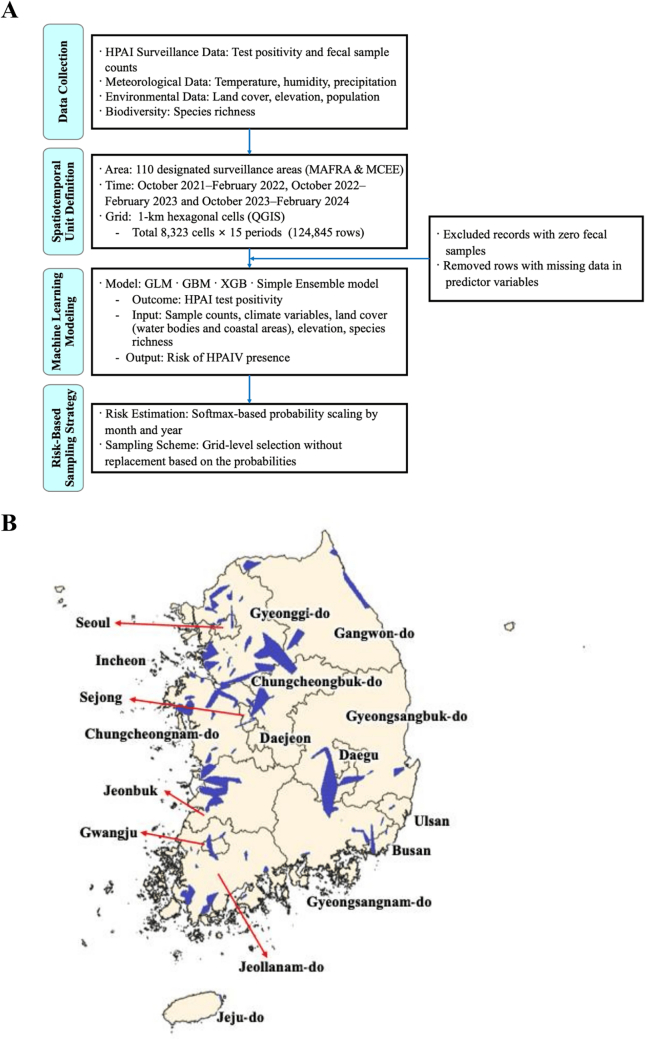
Overview of the study design. (A) Flowchart of the overall study design. (B) Study area: target prediction polygons highlighted in blue were divided into 1-km edge-length hexagonal grids (*n* = 8323). (For interpretation of the references to colour in this figure legend, the reader is referred to the web version of this article.)

This study aimed to optimize fecal surveillance site selection by prioritizing high-risk areas for sampling. To achieve this objective, we developed machine learning (ML) models to predict the risk of avian influenza virus (AIV) detection in fecal samples (Fig. 1). The study area consisted of wild bird fecal surveillance areas designated by the Ministry of Agriculture, Food and Rural Affairs (MAFRA) and the Ministry of Climate, Energy and Environment (MCEE). A total of 110 surveillance areas were included in the analysis and subdivided into hexagonal grids with a 1-km edge length, resulting in 8323 grids. The risk of AIV detection was defined as the probability that AIV-positive fecal samples were detected within a surveillance site, reflecting grid-level spatial occurrence. The study period spanned three winter surveillance seasons: October 2021 to February 2022, October 2022 to February 2023, and October 2023 to February 2024.

### 2.2 Data acquisition and preprocessing

The dependent variable in this study was the detection of AIV in fecal samples. Predictor variables included meteorological factors, remote sensing data, species richness, and sample size (Table 1). These predictors were selected based on their documented associations with HPAI spread in wild birds. All predictors were preprocessed to align with the predefined hexagonal grid structure and subsequently merged into a working dataset for analysis.

**Table 1:** Data utilized in this study.

Predictor	Spatial Resolution	Temporal Resolution	Data source
Sample size	Point-level	Monthly	MAFRA, MCEE
Mean temperature	Point-level	Monthly	ASOS
Mean humidity	Point-level	Monthly	ASOS
Mean precipitation	Point-level	Monthly	ASOS
Elevation	1-km pixel	NA	EOSDIS
Landcover	500-m pixel	Previous year	EOSDIS
Population	1-km pixel	NA	EOSDIS
Richness of Species	100-m pixel	Monthly	Yoo D-S et al., 2022

Data sources for sample size, meteorological factors, remote sensing data, and species richness were provided by MAFRA and MCEE, the Automatic Synoptic Observation System, and NASA's Earth Observing System Data and Information System (EOSDIS).

Climatic variables, including temperature, humidity, and precipitation, were incorporated due to their established influence on both the virus survival and migratory patterns of wild birds [17], [18], [19]. Since wild birds frequently stop at water bodies, where influenza viruses can persist under favorable environmental conditions, water-related land cover variables were included [20], [21]. Human population density was used as a proxy for anthropogenic pressure, as human activity has been shown to negatively affect species diversity and wild bird population size [22], [23], [24]. Elevation was considered because of its negative association with HPAI outbreaks [17], [18]. Species richness was incorporated as an indirect ecological indicator of avian diversity and potential transmission complexity [25].

Data on the dependent variable and sampling size were provided by MAFRA and MCEE. These data covered the period from October to February for each of the three surveillance years (October 2021–February 2022, October 2022–February 2023, and October 2023–February 2024). All positive AIV cases were included regardless of subtype or pathogenicity classification, and positivity was confirmed according to the Standard Operating Procedures for avian influenza [53]. This approach was adopted because ecological hotspots of AIV detection are likely to overlap with areas at risk for HPAI virus introduction and outbreaks. Previous studies have reported reassortant viruses derived from both highly pathogenic and low pathogenic avian influenza strains, suggesting ecological and epidemiological association between HPAI viruses and LPAI viruses. In other words, these findings indicate that both virus types may share with similar migratory wild bird hosts and environmental conditions and habitat characteristics. Fecal surveillance data, including sampling points and positive cases, were initially provided at the point level. These points were projected onto the predefined grid layer, and the number of sampling points within each grid cell was counted separately. These data were summarized in the Supplementary Material. Positive cases were transformed into a binary outcome variable, with grid cells labeled as “1” if they contained at least one positive point and “0” otherwise. This binary variable was used as the dependent variable, while the number of sampling points per grid cell was included as a predictor.

Average temperature, relative humidity, and precipitation were included as meteorological predictors. Raw data were obtained from the Automated Synoptic Observing System operated by the Korea Meteorological Administration [26], which provides monthly averages along with the geographic coordinates of each monitoring site. Data were collected for the period October–February from 2021 to 2024. Ordinary kriging was applied to interpolate continuous spatial surfaces for each variable, after which interpolated values were extracted for each grid cell and averaged within the cell.

Land cover data were obtained from the MODIS Terra+Aqua Land Cover Type Yearly L3 product provided by NASA [27]. The “LC_Type1: Annual International Geosphere-Biosphere Programme (IGBP)” layer was used, which classifies global land cover into 17 categories in raster format at 500-m resolution. Each pixel was assigned a categorical value (1–17) representing the land cover type at that location. For each grid cell, the number of pixels in each category was calculated and converted to proportions. For the study period, the corresponding land cover datasets were from 2020, 2021, 2022, and 2023. Temporal alignment was achieved by using land cover data from the preceding year; for example, 2020 data were assigned to 2021 observations. This approach accounted for potential lag effects of land cover change on wild bird habitats and disease dynamics, while ensuring consistent predictors across study years and maximizing use of available datasets. Of the 17 categories, only two—water bodies and coastal areas—were included as predictors in the model.

For elevation, the GEDI L3 Gridded Land Surface Metrics, Version 2 dataset provided by NASA was used [28]. This raster dataset has a spatial resolution of 1 km and provides continuous elevation values for each pixel. The median elevation within each grid cell was used as the representative value. As the dataset covers the period from 18 April 2019 to 22 March 2023, the same elevation values were applied across the entire study period (2021–2024).

For the human population, the Gridded Population of the World, Version 4 dataset was used, which provides raster data at a 1-km resolution [29]. Population values were extracted from all raster pixels within each grid cell and averaged to obtain a single value per cell. Due to the absence of annual population data, this value was assumed to remain constant throughout the study period (2021–2024), and the same dataset was applied to all years.

Species richness was operationally defined as the sum of binary values indicating the presence of each species [30]. To generate this variable, habitat suitability data for eight species (*Anas formosa*, *Anas acuta*, *Anser albifrons*, *Anas crecca*, *Aix galericulata*, *Anas platyrhynchos*, *Anser fabalis*, and *Anas poecilorhyncha*) were used based on predictions from a MaxEnt model [26]. These datasets contain monthly habitat suitability values (October–February) for each species in raster format. Binary values were assigned by determining whether the monthly suitability exceeded the species-specific median. Finally, values were summed across species to calculate the species richness for each grid cell.

### 2.3 Model fitting and evaluation

Four ML algorithms were developed and evaluated to predict the risk of AIV detection in fecal samples: three individual models—logistic regression (general linear model; GLM), gradient boosting machine (GBM), and extreme gradient boosting (XGB)—and a simple ensemble model that averaged their predictions. Grid cells with zero samples were excluded because they do not contribute to model training due to the absence of labels, resulting in a final dataset (*n* = 1946). All predictor variables were standardized using z-score normalization. The dataset was randomly partitioned into training (80%) and testing (20%) sets. Data for each case were randomized into only one of these sets regardless of temporal order. For hyperparameter tuning of the GBM and XGB, the original training set was further partitioned into sub-training and validation sets with a 7:1 ratio. Random search with root mean square error (RMSE) as the evaluation metric was then used to obtain the best-performing models. In the ensemble model, predictions from the three individual models were averaged. To interpret the relative influence of predictor variables on model predictions, regression coefficients for GLM were calculated using the summary() function, while feature importance analysis for GBM and XGB was conducted using the summary.gbm() and xgb.importance() functions, respectively.

Model performance was assessed on the test set using accuracy, sensitivity, specificity, receiver operating characteristic (ROC) curve, and area under the curve (AUC) values. The optimal classification threshold was defined as the point maximizing the Youden index, which assigns equal weight to sensitivity and specificity [31]. All analyses were performed in the R environment using the *caret*, *gbm*, *xgboost*, and *pROC* packages.

### 2.4 Surveillance strategy design

We developed a sampling strategy based on the estimated risk of AIV detection in fecal samples for each grid cell by month. First, the model which showed the highest AUC value was selected as the best-performing model, and the sampling size for all grid cells was set to the median sample size of the final dataset. Second, risk values were estimated for the grid cells using the predictor variables, with the sample size variable replaced by the new sampling size. Third, all risk values were grouped by year and month and transformed using the SoftMax function to ensure that the probabilities across all grid cells within each period summed to 1 [32]. Finally, stratified resampling without replacement for the grid cells by year and month was performed using those normalized values as probabilities, thereby identifying optimal sites for fecal-based surveillance to serve as an EWS for poultry. To evaluate the practical applicability of this approach, we compared the prioritized high-risk sites identified by the model with the current surveillance sites and visualized the differences between them.

## 3 Results

### 3.1 Model performance

**Fig. 2:**
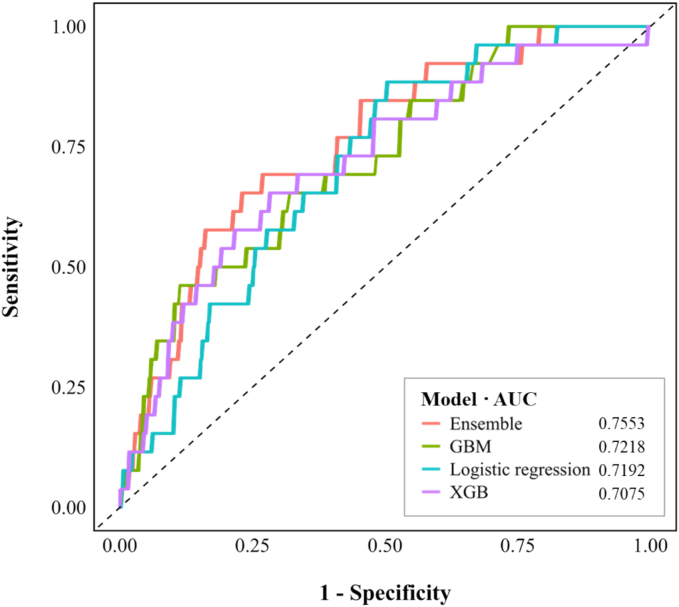
Receiver operating characteristic curves for the four models. ROC–AUC curves of the four models derived from internal validation analysis. The models were ranked from highest to lowest by AUC as follows: ensemble, GBM, GLM, and XGB.

The predictive performance of the four ML models was evaluated based on accuracy, sensitivity, specificity, and AUC values using internal validation with a random split of the dataset. The results for each model are reported in Table 2, and ROC curves are presented in Fig. 2. The GLM achieved the highest sensitivity, indicating strong ability to detect positive cases; however, its accuracy was only slightly above 0.5 due to the specificity being the lowest. The GBM demonstrated the lowest sensitivity but achieved higher specificity and accuracy, ranking second overall. The XGB model showed well-balanced performance across accuracy, sensitivity, and specificity. The ensemble model consistently performed well across all evaluation metrics and achieved the highest AUC among the four models.

**Table 2:** Results of the internal validation procedures for the four models.

Model	Accuracy	Sensitivity	Specificity	AUC
Logistic regression	0.5232	0.8846	0.4972	0.7075
GBM	0.8582	0.4615	0.8867	0.7218
XGB	0.7139	0.6538	0.7182	0.7192
Ensemble	0.7629	0.6538	0.7707	0.7553

Accuracy, sensitivity, specificity, and ROC–AUC values were obtained from internal validation. All values were calculated at the optimal threshold determined by the Youden index.

### 3.2 Risk index mapping

**Fig. 3:**
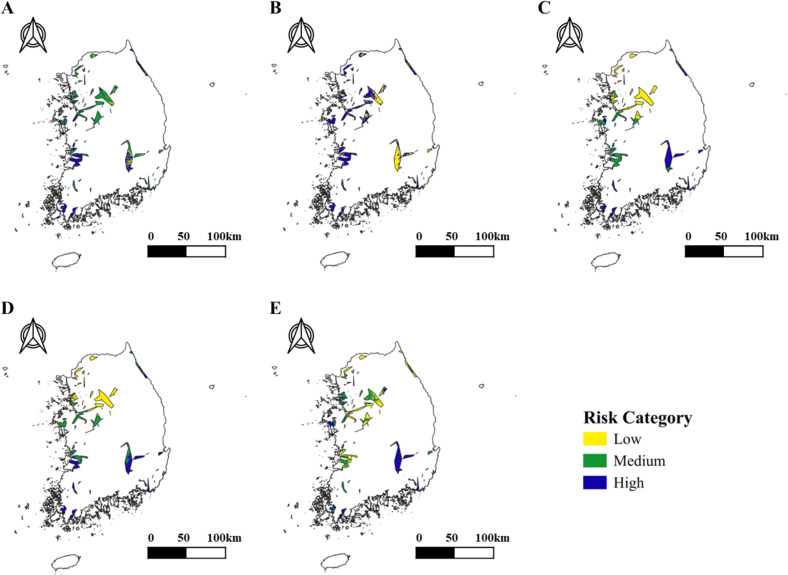
Monthly average risk of AIV detection in feces predicted by the ensemble model. Risk maps were generated using the best-performing ensemble model (A: October, B: November, C: December, D: January, E: February). Colors represent risk categories (low, medium, high).

Monthly risk maps were generated for each model. The map produced by the best-performing model, the ensemble, is presented in Fig. 3, while the maps from the other models are provided in the Supplementary Fig. 1–3. To define monthly risk levels, average risk values were divided into three quantiles (1/3 and 2/3) and classified as low, medium, or high risk. Across most months, Jeollanam-do and the western coastal region consistently exhibited high risk. The outskirts of Gyeonggi-do tended to show high risk, with the exception of December and January. Gyeongsang-do, including Daegu, demonstrated consistently high risk except in November. In addition, Gangwon-do displayed elevated risk from October through December.

### 3.3 Feature importance across models

Feature importance results are summarized in Table 3. Meteorological factors and sample size were consistently influential, with the exception of the XGB model. In the GLM, temperature and sample size had positive coefficients, indicating that higher temperature and a larger number of samples were associated with increased risk. In ML-based models, the ranking of feature importance varied slightly, but meteorological factors consistently ranked among the most important predictors. GBM indicated that sample size had a particularly high influence, similar to the results of the GLM.

**Table 3:** Feature significance and importance across models.

Predictor variable	Logistic Regression	GBM	XGB
Humidity	0.0742	3	3
Precipitation	−0.1936	4	2
Temperature	0.4909*	1	1
Population	−0.0415	6	4
Elevation	−0.1576	5	6
Water bodies / Coastal areas	0.0413	8	8
Species richness	−0.0884	7	7
Sample size	0.3508*	2	5

For GLM, “+” and “−” indicate positive and negative associations, respectively, and “*” denotes statistical significance at *p* < 0.01. GBM and XGB models present variable importance rankings, with rank 1 representing the most influential predictor.

### 3.4 Comparison between current and proposed sampling approaches

**Fig. 4:**
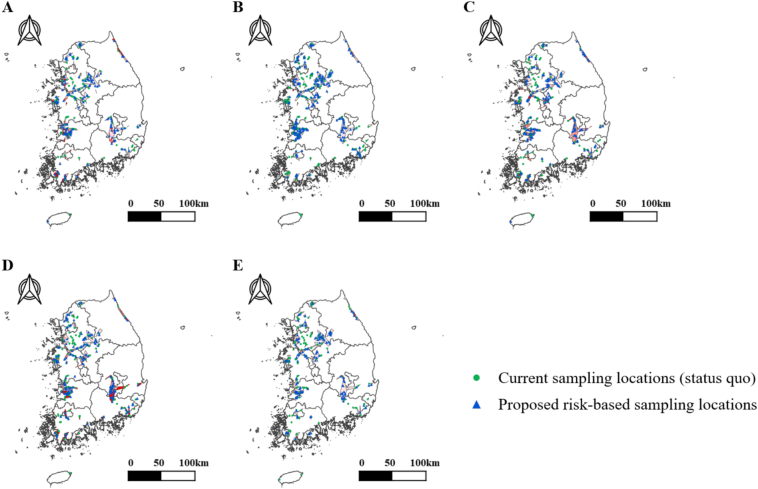
Comparison between current and proposed sampling sites. Monthly risk maps from October 2023 to February 2024 were generated using the ensemble model. Green circles represent actual sampling sites, while blue triangles indicate risk-based sampling sites selected according to monthly predicted risk. (For interpretation of the references to colour in this figure legend, the reader is referred to the web version of this article.)

Based on the monthly predicted risk from October 2023 to February 2024, hypothetical sampling sites were selected using the proposed strategy. The number of samples per month was set equal to the actual number of samples collected during the corresponding month. For comparison, actual sampling sites and risk-based sampling sites were overlaid on the same risk map (Fig. 4). Both strategies showed a tendency to concentrate sampling along the west coast and in the outskirts of Gyeonggi-do. However, despite consistently high predicted risk in Gangwon-do, relatively few actual samples were collected there, whereas the risk-based sampling strategy actively prioritized this region. Furthermore, while some actual sampling was conducted in Gyeonggi-do, fewer sites in this area were suggested under the risk-based approach.

The risk values estimated by the four ML models (GLM, GBM, XGB, and ensemble) are provided in the Supplementary Material, and detailed hyperparameter settings for GBM and XGB are provided in Supplementary Table 1 and 2.

## 4 Discussion

In this study, we proposed an optimal site selection strategy for fecal surveillance through ML-based risk assessment. All models demonstrated reliable predictive performance, with the ensemble model performing best. Using the risk values generated by this model, we identified optimal surveillance sites, which showed a clear divergence from the currently established sites. These findings suggest that the proposed site selection strategy could substantially enhance the existing surveillance system as an EWS.

The ensemble model achieved an AUC of 0.7553, with accuracy, sensitivity, and specificity of 0.7629, 0.6538, and 0.7707, respectively. In general, an AUC greater than 0.7 is considered indicative of reasonable predictive capability. Previous studies on infectious disease risk prediction, including malaria and dengue fever, have demonstrated that ML models achieve higher predictive performance than conventional statistical approaches [33], [34], [35], [36], [37]. In particular, ensemble models often outperform single algorithms [36], [37], [38], [39], [40]. Our results were consistent with these findings, suggesting that the ensemble approach provides a robust framework for predicting the risk of AIV detection based on fecal sampling data.

In the ensemble model's monthly predictions, high-risk areas were concentrated along the west coast from November to January, whereas the risk was more geographically dispersed in October and February [42], [43], [44]. Most migratory birds entering South Korea are known to overwinter in regions near the west coast, and this ecological context supports the observed concentration of high-risk areas during the winter months, reflecting migratory and overwintering patterns [45]. Daegu and parts of Gyeongsangbuk-do were also repeatedly identified as high-risk regions. This pattern aligned with previous studies reporting an expansion of high-risk areas from the Midwestern region toward the southeast along the Nakdong River [30].

Predictor variables, such as meteorological factors, sample size, population, and elevation, were identified as important contributors to model performance. Among these, temperature consistently emerged as the most important variable across all models, consistent with previous studies. In contrast to earlier research, humidity, precipitation, and elevation were not significant in the GLM, likely due to environmental differences between our study and others. In particular, regarding elevation, the limited variance resulting from restricting the study area to specific surveillance sites likely masked its significance in the GLM. However, their importance was confirmed in the ML models, which can capture nonlinear relationships among predictors. Population was included as a proxy for indirect human influence and demonstrated moderate importance.

A spatial inconsistency was observed between current sampling sites and the proposed risk-based sampling sites. For instance, Gyeonggi-do had a large number of collected samples despite its relatively low predicted risk, whereas Gangwon-do showed the opposite trend. These discrepancies suggest that current sampling strategies may be driven more by administrative factors, such as accessibility, than by actual risk [46]. In practice, sampling locations are often determined based on previous case reports, expert opinions regarding suspected transmission areas, convenience of access, or a combination of these considerations, which may reduce the effectiveness of surveillance [47].

While this study is expected to make a practical contribution by establishing a surveillance system grounded in quantitative risk prediction, several limitations must be acknowledged. First, there remains room for improvement in the variables used to build the models. For example, previous studies have highlighted associations between road networks and HPAI spread [21], [41], [48], and ongoing environmental changes are altering migratory bird pathways [49], [50]. In addition, AIV-positive cases regardless of subtype and pathogenicity were used for model development because the number of confirmed HPAI-positive cases was limited and detailed virological information was unavailable for all positive cases. The predictive models can be further refined by continuously accumulating and incorporating subtype- and pathogenicity-confirmed HPAI virus surveillance data, along with additional environmental and ecological variables. Second, despite broad temporal and spatial coverage, the dataset included only 1946 samples. This relatively small sample size may constrain the generalizability of the models. Moreover, because the analysis was limited to sites designated by MAFRA and MCEE, high-risk areas outside this surveillance system may have been missed, potentially creating spatial blind spots. To minimize this issue, further review of surveillance site placement through consultation among experts and field practitioners, will be necessary. Lastly, uncertainties inherent to the data itself should be considered. In particular, climatic and species richness variables relied on interpolation-based estimates, which may lead to estimation errors. To address these limitations, future research should incorporate additional explanatory variables related to evolving environmental conditions and wild bird habitats and should utilize continuously collected data to regularly update predictive models [51].

Nevertheless, this study emphasizes the practical necessity of improving the current surveillance system for HPAI through a risk-based approach. By comparing optimal surveillance sites with current sampling sites, the proposed strategy demonstrates its potential to enhance the EWS for poultry and improve the efficiency of resource allocation. Moreover, the developed framework could be extended to the surveillance of other infectious diseases characterized by dynamic ecological and epidemiological patterns [52]. Ultimately, we expect that the feces-based surveillance system developed in this study will contribute significantly to strengthening preventive policies for HPAI as well as other infectious diseases.

## 5 Conclusions

This study estimated the risk of AIV detection from fecal samples using diverse categories of variables and proposed a quantitative surveillance strategy. Among the models tested, the ensemble model demonstrated the best performance (AUC = 0.7553), and inconsistencies were identified between current surveillance sites and risk-based sites. These findings indicate that the proposed strategy can enhance surveillance efficiency by prioritizing sites for fecal sampling. Such an approach can support evidence-based decision-making for sampling site selection and resource allocation in national HPAI control policies, thereby contributing to the early prevention and mitigation of HPAI spread to domestic poultry. Moreover, the proposed framework can be continuously improved through ongoing data accumulation and model refinement. It also has potential applicability to the surveillance of emerging animal diseases and other zoonoses. Ultimately, this approach may contribute to strengthening public health within the One Health framework.

## CRediT authorship contribution statement

**SunMin Kim:** Writing – review & editing, Writing – original draft, Visualization, Validation, Formal analysis, Data curation. **Hyunjun Cho:** Project administration, Conceptualization. **Hyesung Jeong:** Project administration, Conceptualization. **Dae Sung Yoo:** Project administration, Methodology. **Kyuyoung Lee:** Project administration, Methodology. **Hu Suk Lee:** Project administration, Methodology. **Kyung-Duk Min:** Writing – review & editing, Validation, Project administration, Methodology, Formal analysis, Data curation.

## Ethics approval

Not applicable.

## Declaration of competing interest

The authors declare that they have no known competing financial interests or personal relationships that could have appeared to influence the work reported in this paper.

## Data Availability

The datasets used and/or analyzed during the current study are available from the corresponding author on reasonable request.
