# Supplementary material for: Optimizing wild bird fecal surveillance sites for HPAI through risk estimation using machine learning algorithms
Source: One Health. 2026 May 27;22:101457. doi: 10.1016/j.onehlt.2026.101457 (PMC13253128; doi:10.1016/j.onehlt.2026.101457)
Supplement: Supplementary file 1 — Supplementary material 1 [file mmc1.docx]

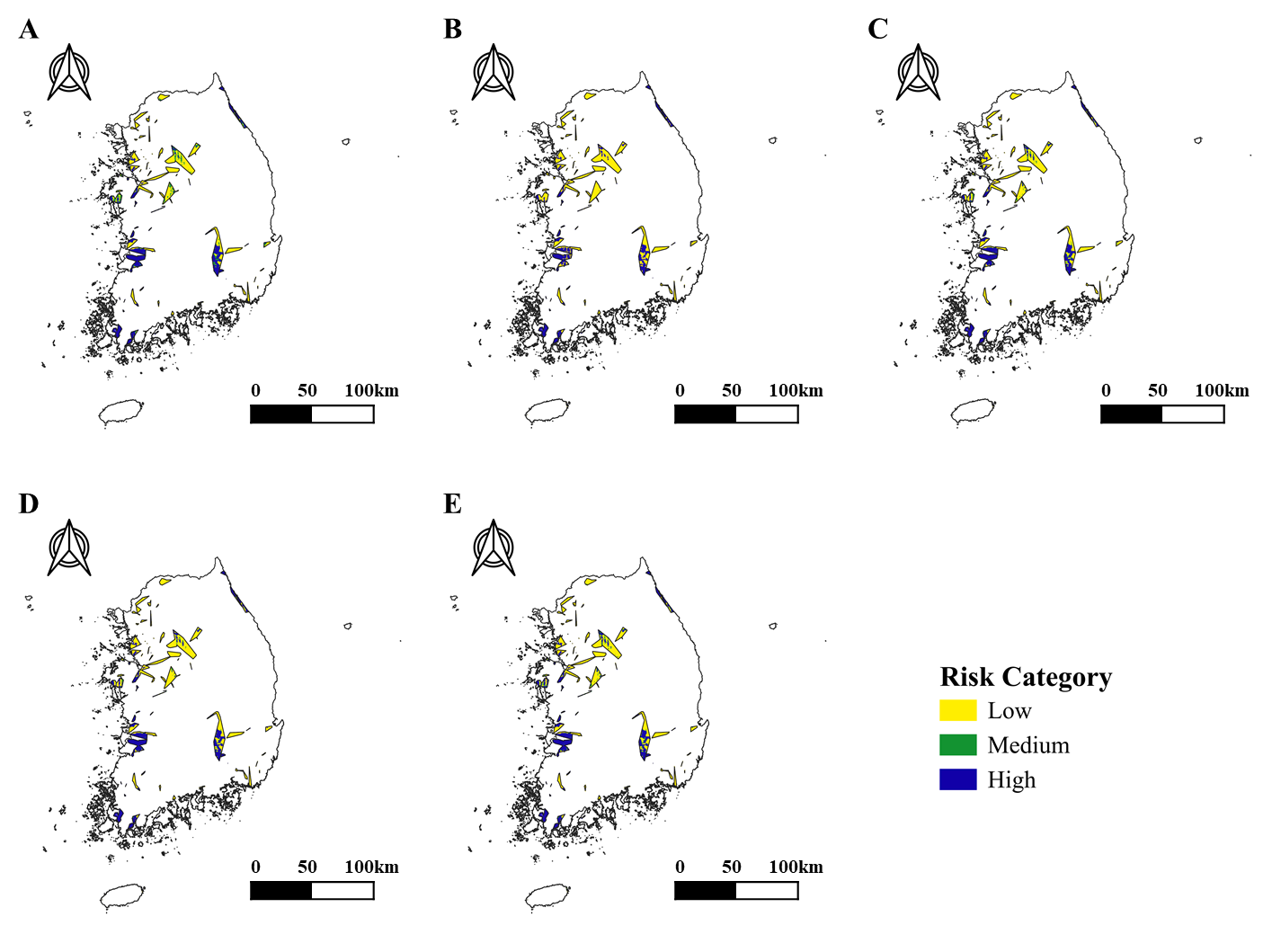


**Supplementary Figure 1.** Monthly average risk of HPAIV presence in feces predicted by the logistic regression model.

Note. Risk maps were generated using the best-performing ensemble model (A: October, B: November, C: December, D: January, E: February). Colors represent risk categories (low, medium, high).


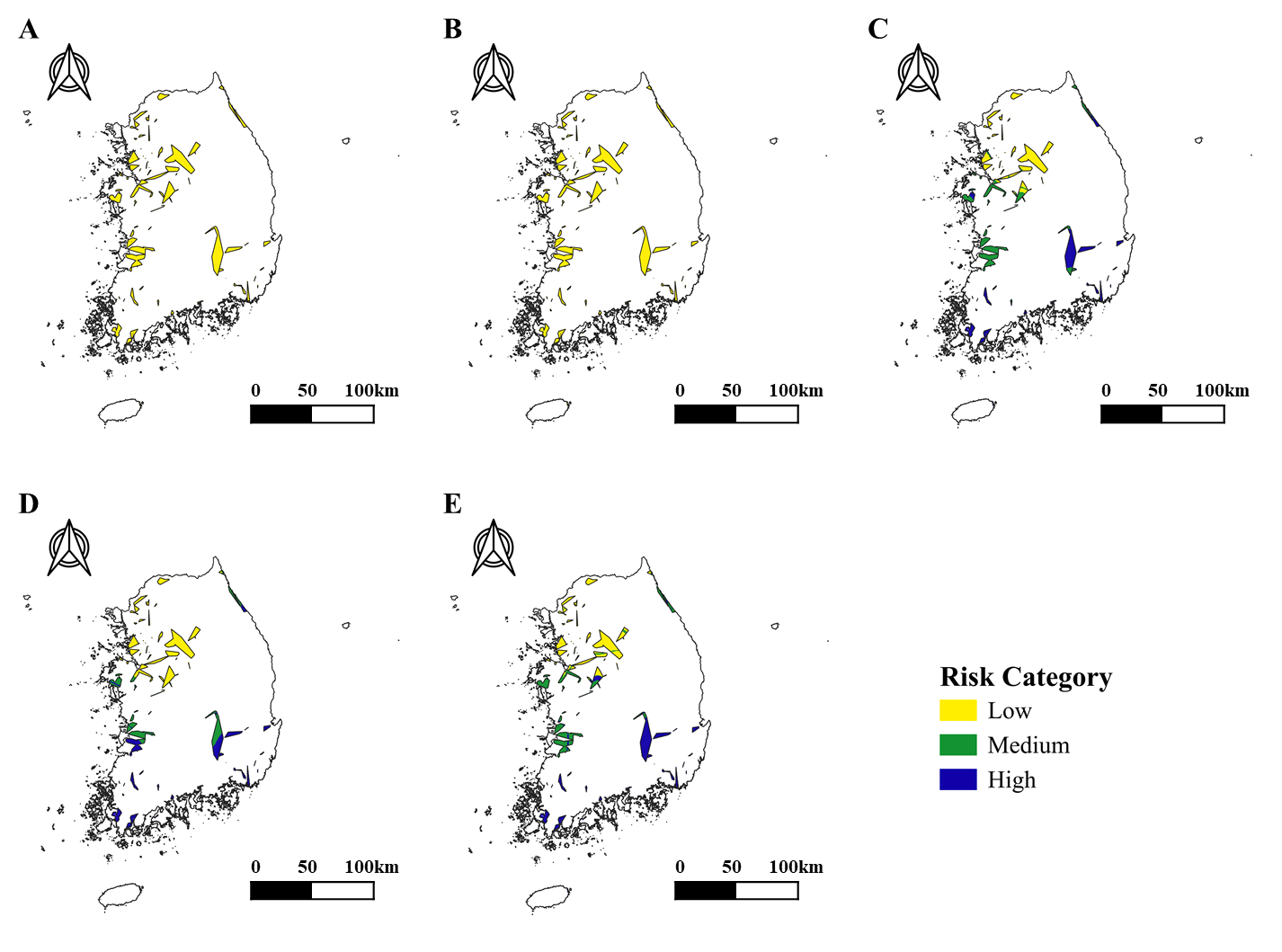


**Supplementary Figure 2.** Monthly average risk of HPAIV presence in feces predicted by the gradient boosting model.

Note. Risk maps were generated using the best-performing ensemble model (A: October, B: November, C: December, D: January, E: February). Colors represent risk categories (low, medium, high).


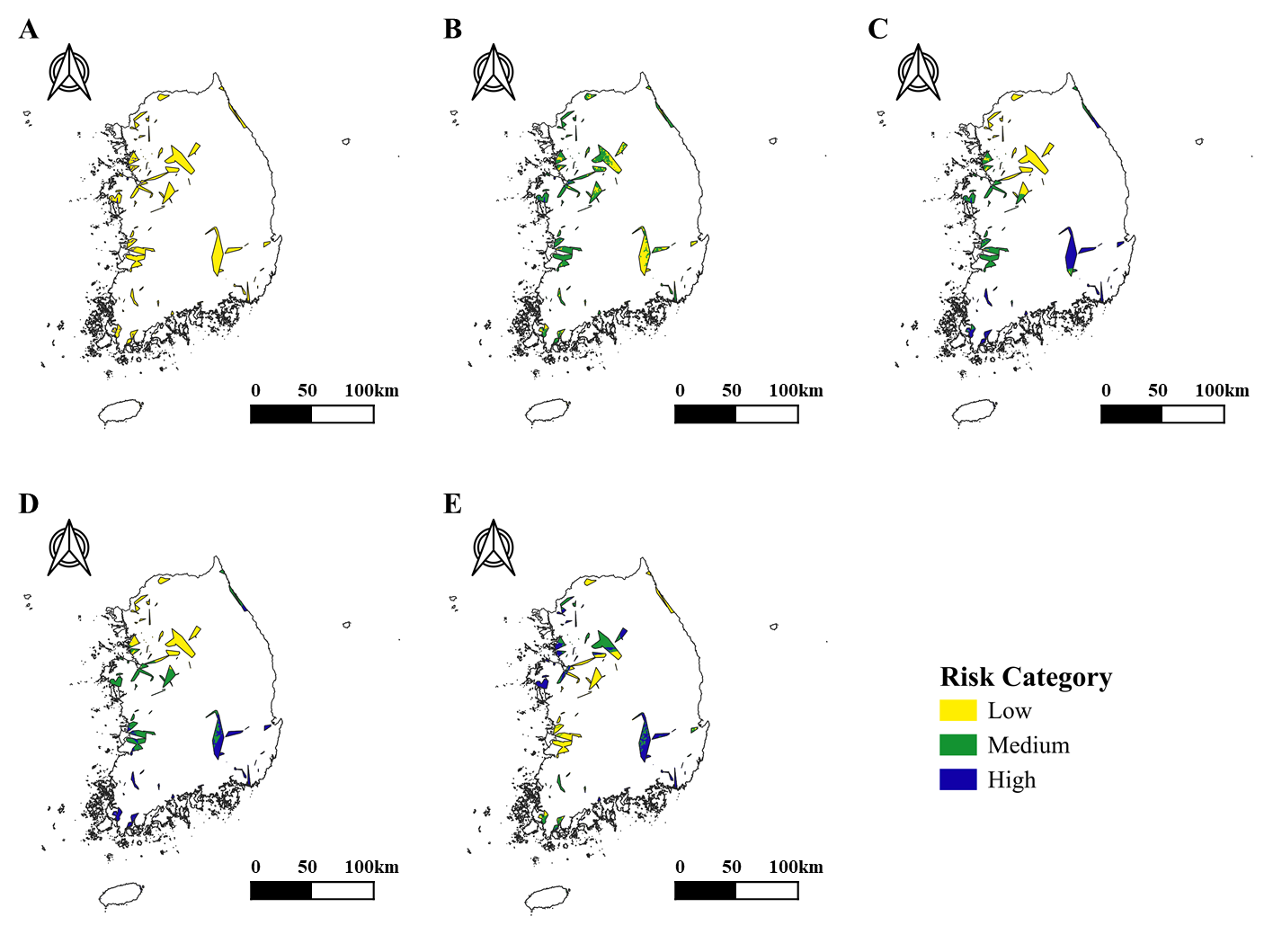


**Supplementary Figure 3.** Monthly average risk of HPAIV presence in feces predicted by the extreme gradient boosting model.

Note. Risk maps were generated using the best-performing ensemble model (A: October, B: November, C: December, D: January, E: February). Colors represent risk categories (low, medium, high).

**Supplementary Table 1.** Range of tested hyperparameter values for GBM and XGB

| **Classifier** | **Parameter** | **Description** | **Tested Parameter Ranges** |
| --- | --- | --- | --- |
| GBM | n.trees | Number of trees to be generated | 1:8000 |
|  | interaction.depth | Maximum depth of the tree | 3, 7, 10 |
|  | shrinkage | Learning rate for boosting | 0.01, 0.05, 0.1 |
|  | n.minobsinnode | Minimum samples in each leaf node | 5, 7, 10 |
|  | bag.fraction | Subsample ratio of rows | 0.65, 0.8, 1.0 |
| XGB | max.depths | Maximum depth of the tree | 7, 10 |
|  | etas | Learning rate for boosting | 0.01, 0.001 |
|  | n_threads | Number of cores to be used for parallel processing successive tree | 2 |

Note. All names listed in the ‘Parameter’ column correspond to built-in option names of each model in R.

**Supplementary Table 2.** Hyperparameters for each machine learning model

| **Parameter** | **Description** | **A. GBM** | **B. XGB** |
| --- | --- | --- | --- |
| A. shrinkage  B. etas | Learning rate for boosting | 0.1 | 0.01 |
| A. interaction.depth  B. max_depth | Maximum depth of the tree | 3 | 7 |
| A. n.trees  B. nrounds | Number of trees to be generated | 32 | 1000 |
| A. bag.fraction | Subsample ratio of rows | 0.65 | null |
| A. n.minobsinnode | Minimum samples in each leaf node | 5 | null |
| B. n_threads | Number of cores to be used for parallel processing successive tree | null | 2 |
| Evaluation metric | Performance metric used for model selection | RMSE | logloss |

Note. All names in the ‘Parameter’ column correspond to the built-in argument names of each model in R. Those marked ‘A’ refer to GBM, ‘B’ to XGB, and those without any mark are common to both.
